# Nasal Polypi

**Published:** 1887-04

**Authors:** 


NASAL POLYPI.—Dr. William R. Bell, *Canada Medical Record,* describes a new, painless and simple method of removing nasal polypi. His patient is instructed to blow strongly through the affected nostril while he closes the other with his fingers. This brings the polypus down so that it can be seen. He then injects into the tumor, with a hypodermic syringe, fifteen or twenty minims of a solution of tannin in water (twenty grains to a fluid drachm). In a few days the tumor shrivels, dries up and comes away without trouble or pain, the patient usually removing it with his fingers or by blowing his nose.

